# Seawater Corrosion Resistance of Duplex Stainless Steel and the Axial Compressive Stiffness of Its Reinforced Concrete Columns

**DOI:** 10.3390/ma16237249

**Published:** 2023-11-21

**Authors:** Zhenhua Ren, Lizheng Fang, Hui Wang, Peng Ding, Xiantao Zeng

**Affiliations:** 1 Hunan Provincial Key Laboratory of Intelligent Disaster Prevention-Mitigation and Ecological Restoration in Civil Engineering, Hunan Institute of Engineering, Xiangtan 411104, China; zhhren81@163.com (Z.R.); fanglizheng9507@hotmail.com (L.F.); 18175838652m@sina.cn (H.W.); 2 China Construction Science & Technology Group Co., Ltd., Beijing 100195, China; dingpeng20@tsinghua.org.cn

**Keywords:** duplex stainless steel, seawater simulation, polarization curve, stiffness analysis

## Abstract

In order to explore the corrosion resistance of duplex stainless steel under seawater corrosion and the compressive stiffness of its reinforced concrete columns, this study first performed seawater corrosion resistance tests on HRB400 ordinary steel rebar and S32205 duplex stainless steel rebar. The effect of the corrosion product film on the corrosion behavior was investigated through polarization curve tests and electrochemical impedance spectroscopy tests. The results showed that the corrosion rate of S32205 duplex stainless steel in a seawater environment was approximately 1/15 that of the HRB400 ordinary steel rebar. The anodic polarization curve of duplex stainless steel rebars exhibited a greater slope than that of carbon steel rebars. In the simulated seawater environment, the corrosion rate of these two kinds of steel bars showed different trends. The corrosion rate of ordinary steel bar HRB400 first decreased and then increased, while that of duplex stainless steel S2205 increased steadily. Furthermore, 18 short concrete columns reinforced with ordinary and duplex stainless steel rebars were subjected to the axial compression test and stiffness analysis; the stiffness of the short columns was calculated from the test data. The theoretical values agreed with the test values, with a stiffness calculation error of less than 5%.

## 1. Introduction

Duplex stainless steel is a resource-saving, high-performance steel product, and its production and application are consistent with the future development of stainless steel [1,2,3,4,5,6,7]. The development of ocean-related resources has always been a research hotspot. Duplex stainless steel has good chloride corrosion resistance, pitting resistance, high mechanical strength, and low manufacturing costs. On this basis, it is feasible to replace steel structural members with poor corrosion resistance and commonly used high chromium-nickel stainless steel structural members with duplex stainless steels. It can be expected that duplex stainless steel will have significant application prospects in cross-sea engineering, coastal engineering, submarine engineering, offshore drilling platforms, offshore wind power engineering, shipbuilding, and coastal area weaponry [8,9,10,11]. 

The ocean is a highly corrosive disaster environment, with various materials susceptible to deterioration and damage. The total annual economic loss caused by corrosion accounts for 2–4% of the world’s GDP, of which the loss of marine corrosion accounts for approximately 1/3 of the total corrosion [12]. Mehta [13] pointed out that corrosion of reinforcing steel is the most important factor affecting the durability of marine concrete. Guzmán-Torres et al [14] This study evaluates the performance of real-time object detection using the You Only Look Once, Version 3, algorithm to detect corrosion damage in concrete structures. The structure of YOLOv3 is based on a complex but effective convolutional neural network; two training stages are established with data to improve the model accuracy. The test results show that the concrete corrosion detection is satisfactory using medium-resolution and high-resolution training images for transfer learning. Sheetal [15]. In this paper, the basic principles and research progress of six electrochemical methods (linear polarization resistance, electrochemical noise technology, scanning ion selective electrode technology, electrochemical Shi Ying crystal microbalance technology, scanning vibrating electrode technology, and electrochemical frequency modulation) are discussed. These technologies can be used for corrosion detection and diagnosis to better understand the research progress in this field [16]. 

In this study, tensile tests were carried out on corroded steel bars in buildings exposed to natural chloride corrosion and A706 corroded steel bars obtained from artificial corrosion by the impressed current method. According to the test results, a reduction factor was put forward that relates the tensile properties of naturally corroded steel bars and artificially corroded steel bars with the corrosion quality loss. In addition, the reduction factors of natural and artificial corrosion steel bars in previous studies were collected. Compared with previous research, the reduction coefficient of steel bars corroded by chloride is usually greater than that of carbonation. Zhu [17] simulated the high temperature, high pressure, and high chloride ion conditions of the deep-sea hydrothermal area. The electrochemical properties and passivation film characteristics of 2205 duplex steel were evaluated, revealing the influence of temperature on its corrosion behavior. Sri et al. [18] compared the crevice corrosion behavior of three duplex stainless steels (2101, 2205, and 2507) in seawater containing 200 ppm hypochlorite. Dong [19] used a 2205 duplex stainless steel plate as a research object to study its electrochemical corrosion behavior in NaCl solutions of different temperatures and concentrations. These studies revealed the corrosion behavior and characteristics of 2205 duplex stainless steel materials in different environments. However, the corrosion rate has not been investigated in comparison with other types of steel rebars to demonstrate the advantages of 2205 duplex stainless steel in marine environments. In addition, the understanding of the corrosion behavior of duplex stainless steel rebars remains incomplete.

Carbon steel remains the dominant reinforcing steel for reinforced concrete structures [20]. In a marine environment with high temperature, humidity, and salt air, the service life of carbon steel is severely limited. Corrosion-resistant steel rebar is the last barrier to prevent premature failure of reinforced concrete members or structures caused by steel rebar corrosion [21]. Previous studies demonstrated that duplex stainless steel, as a stainless steel rebar, has better mechanical properties and corrosion resistance than ordinary steel rebars [22,23,24,25]. Replacing ordinary steel rebar with stainless steel rebar can increase the resistance of the structure to chloride attack by more than one order of magnitude [26,27,28,29,30]. Stainless steel rebar is characterized by oxidation resistance, high-temperature resistance, corrosion resistance, high strength, and a high flexural strength ratio [31,32,33,34,35,36]. Using stainless steel rebar prolongs the service life of the structure and reduces the subsequent repair and maintenance costs, thus lowering the total cost over the life cycle of the structure [37,38,39]. In recent years, stainless steel materials have been widely applied in several practical projects, especially in bridges, retaining walls, and tunnels suffering from strong chemical and seawater erosion [40,41,42,43,44]. For example, the Stonecutters’ Bridge in Hong Kong and the Sheikh Zayed Bridge in Abu Dhabi used 1.4462 stainless steel rebars to reinforce concrete in seawater splash zones and seawater erosion-prone members. In addition, duplex stainless steel rebar was used in the abutment structures of the Hong Kong-Zhuhai-Macao Bridge [45], with a design reference period of 120 years.

With increasing applications of stainless steel rebar, more attention has been paid to the mechanical properties of stainless steel reinforced concrete (SSRC) structures or members. First, the bonding performance and cooperative working performance of stainless steel rebars and concrete were considered. The relevant experimental studies [46,47,48] have proven that stainless steel rebar has good bonding performance with concrete. Second, attention has also been paid to the bending properties, eccentric compression properties, and load-carrying capacity of SSRC members and structures [49,50]. Relevant studies [33,34,36,51,52,53,54] have shown that SSRC structures have good mechanical properties. However, there are only a few theoretical studies on the engineering application of duplex stainless steel rebar. Research on the bonding performance, mechanical properties, damage modes, and corrosion resistance of duplex SSRC structures is also relatively rare.

Based on the above analysis, this paper took the S32205 duplex stainless steel rebar as a research object. The effects of steel rebar corrosion on the concrete-steel rebar bonding interface in simulated seawater solutions were investigated by electrochemical testing and analysis methods; the corrosion behavior in simulated seawater environments was further explored. Second, based on the corrosion of stainless steel rebar by seawater, 18 short concrete columns were cast with ordinary steel rebar (HRB400) and stainless steel rebar (S32205) as longitudinal rebars. The axial compression tests were performed on these concrete columns to study their axial compression bearing capacity with emphasis on their axial compressive stiffness.

## 2. Seawater Corrosion Test on Stainless Steel Rebars and Axial Compression Test on Concrete Columns

### 2.1. Seawater Corrosion Test on Stainless Steel Rebars

#### 2.1.1. Raw Materials and Proportion

In order to simultaneously study seawater corrosion resistance of both materials and the concrete structure, small specimens of S32205 duplex stainless steel rebar, HRB400 steel rebar, and SSRC were prepared. The chemical compositions of S32205 duplex stainless steel rebar and HRB400 ordinary steel rebar are shown in Table 1.

Concrete was prepared with P.O 42.5 silicate cement produced by Hunan Xiangxiang Chengmei Cement Limited Company, 200 mesh quartz sand produced by Shengli Quartz Sand Factory, and 3–6 mm river pebbles. The concrete mix proportion was cement: water: sand: stone = 0.5:1:1.97:3.35, and the concrete compressive strength was 15.41 MPa. The protective layer of concrete provides a certain degree of resistance to diffusion penetration of chloride ions, requiring a long time for the ions to diffuse from the concrete surface to the steel rebar. In order to shorten the time of chloride ion diffusion, the working surface of the rebar was in contact with the concrete during the specimen preparation, with the thickness of the contact surface between them not exceeding 0.5 mm.

Natural seawater is composed of many chemical components. According to a certain ratio of Na^+^, Cl^−^, K^+^, Br^−^, H^+^, $BO_{3}^{-}$, $SO_{4}^{2-}$, $HCO_{3}^{-}$, Ca^+^, $Mg_{2}^{-}$, $Sr_{2}^{+}$, F^−^, and OH^−^, artificial seawater salt with a Cl- concentration of 3.5% or 5% was prepared. Sodium, magnesium, and potassium were mixed at a 3:2:1 ratio, accounting for 90% of the artificial seawater salt, and the remaining 10% consisted of other trace elements. The reagents were of analytical grade, and solutions were prepared using deionized water.

#### 2.1.2. Preparation and Testing of Working Electrode

**Figure 1:**
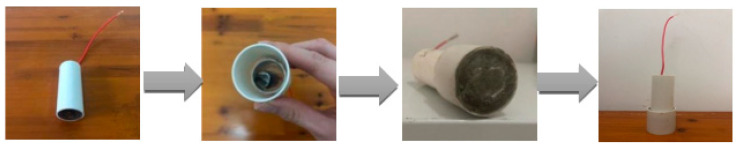
Preparation of working electrode and concrete specimen.

The steel rebar was cut into small short column specimens with a 12 mm diameter and 50 mm length. Before the test, the surface of the specimen was polished with 150, 400, 800, 1200, and 1500 metallographic sandpaper. Then, the specimens were washed with deionized water and anhydrous ethanol and blown dry with cold air. The dried specimens were placed into a PVC tube with an inner diameter of 32 mm and encapsulated with epoxy resin. After chiseling the inside of the PVC tube with an inner diameter of 40 mm and length of 30 mm, it was set on the top surface of the working electrode using a special adhesive and sealed tightly to ensure no water leakage or seepage. Finally, the sand paste was poured into it, and the specimens were fully vibrated and compacted, with the thickness of the specimen protective layer controlled at 10 mm. The specimen preparation process is illustrated in Figure 1.

After curing the mortar specimens of the working electrode in a curing box for 15 d, each was immersed in 500 mL of simulated solution (artificial seawater with Cl- concentration of 3.5% or 5%). The mouth of the beaker was sealed with plastic wrap (to prevent moisture in the air and impurities from entering) and then placed in a water bath at 20 °C for constant heating.

Each group comprised two samples, with a total of 16 samples, all of which were immersed in the simulated environment. The samples were taken out of the beaker at 7, 14, 28, and 42 d, respectively, and the polarization curve and AC impedance spectrum were tested with an electrochemical workstation. To prevent the electrode from damaging the sample during scanning, the same sample was tested by impedance spectrum before the polarization curve.

The main operations of in situ electrochemical testing are as follows: the in situ electrochemical test was performed using an electrochemical workstation model CHI660E. The standard three-electrode system was used, with a saturated calomel electrode (SCE) as the reference electrode and a platinum sheet as the auxiliary electrode. Electrochemical measurements were performed at the same time each day, and open circuit potential (OCP), polarization curve (Tafel), and electrochemical impedance spectroscopy (EIS) were measured separately for each group of specimens. The specific parameters were as follows: the OCP test was performed for 600 s, and the open circuit potential of the 2205 steel sample was tracked by the electrochemical workstation for 25 min to ensure that the corrosion system reached a stable state. Then, the open circuit potential (E_ocp_) measured at 25 min was used as the potential for the electrochemical AC impedance test to assess the electrochemical AC impedance of the sample. The potential interval of the Tafel test was ±300 mV (relative to the OCP), the dynamic polarization curve was scanned at a rate of 0.1 mV/s, and the EIS test was performed at a frequency of 1.0 × (10^−2^–10^4^) Hz with a perturbation of 10 mV for 42 d.

### 2.2. Axial Compressive Stiffness Test of Reinforced Concrete Columns

#### 2.2.1. Design and Preparation of Members

**Figure 2:**
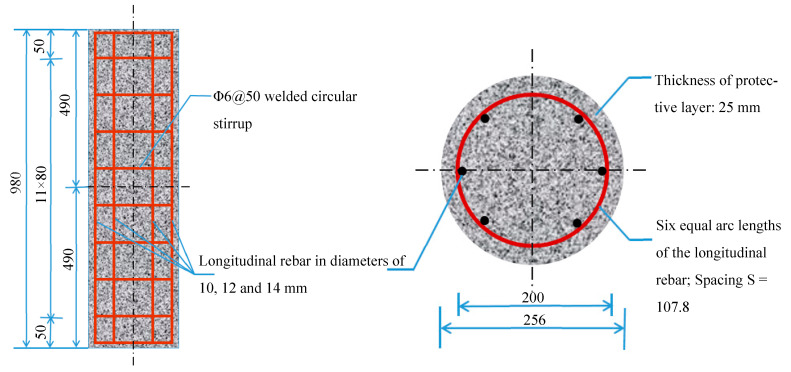
Dimensions and reinforcement of short columns (mm).

A total of 18 short columns were designed for this test, including nine comparison columns. The concrete columns were all circular cross-sections with a diameter of 256 mm and a height of 1000 mm. Ordinary steel rebar HRB400 was selected as the longitudinal rebar of the comparison columns, with diameters of 10 mm, 12 mm, and 14 mm. Each group contained three concrete columns. The stirrup was HRB400 with a diameter of 6 mm and spacing of 50 mm. The longitudinal rebar of the test columns was made of S32205 duplex stainless steel rebar with diameters of 10 mm, 12 mm, and 14 mm. The stirrup was made of S32205 duplex stainless steel rebar, with a diameter of 6 mm and spacing of 50 mm. The geometric dimension and reinforcement of the concrete columns are shown in Figure 2.

**Figure 3:**
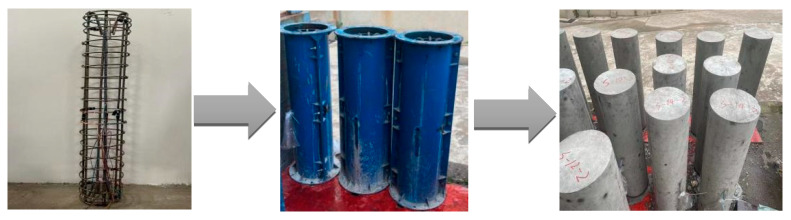
Preparation process of short columns.

The reinforcement skeleton was connected by longitudinal rebar and stirrups. The 10 mm protective layer at the bottom of the short column was made of welded steel rebar, and the strain gauge wires were pulled out by black tape along the longitudinal rebar. C30 concrete was selected as the test specimen. The concrete mix ratio was cement: water: sand: stone = 0.5:1:1.97:3.35, with 37% sand content and P.O 42.5 cement produced by Hunan Xiangxiang Chengmei Cement Limited Company. No admixture was used. The formwork was made of steel and accurately processed according to the dimensions of the steel pipes in the same batch. For smooth demolding, the inside of the pipe was brushed with oil, then the reinforcement cage was accurately placed into the formwork, and the wires were carefully pulled out. In order to prevent leakage, the wire holes on both sides of the pipe were plugged with yellow mud, and black tape was applied to the exterior. Then, the two half-round steel plates were fastened, and the bolts were used to securely assemble the formwork. To prevent the stain gauge wires from soaking and rusting, the wire head was wrapped tightly with black tape, and the wires were covered with two layers of plastic wrap. Afterward, the concrete was poured in two separate steps, with an interval of 18 h between each pouring. In order to avoid the formation of voids and pits in the concrete, vibrating rods were used to vibrate the concrete during pouring. Finally, the column tops were flattened, and standard concrete test blocks were fabricated simultaneously. The removal of molds for short columns can only be performed after 24 h of pouring. The yellow mud and the cement in the wire holes on the steel formwork were first cleaned, followed by the removal of the bolts using an electric wrench. Finally, the formwork was gently cracked open using a crowbar. Constant attention should be paid to the wires during mold removal to prevent them from being pulled out. The preparation process of short columns is illustrated in Figure 3.

#### 2.2.2. Testing and Measurement Methods

**Figure 4:**
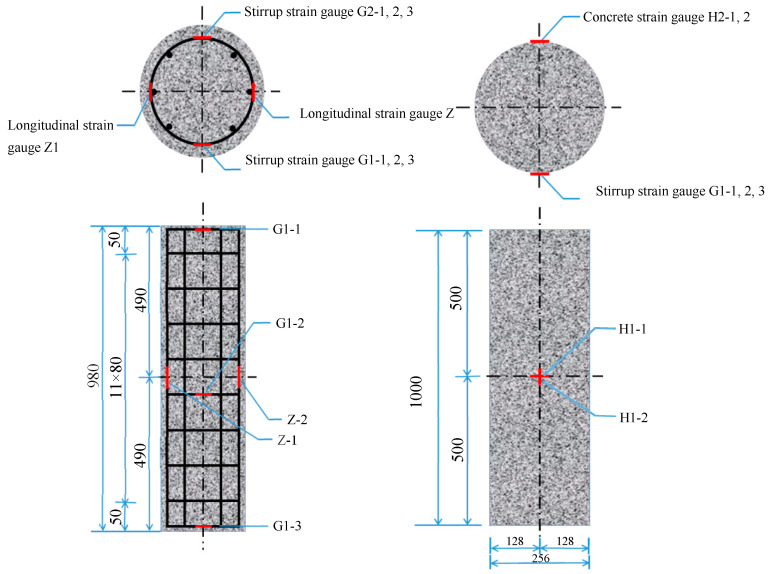
Strain gauge positions.

To measure the stress and deformation of steel rebars, concrete, and steel pipe, strain gauges were attached to specific parts. The resistance strain gauge used in this experiment was 120 Ω. Six strain gauges were evenly attached to the upper, middle, and lower parts of the stirrup in the column. Among the six longitudinal rebars, strain gauges were attached to three, with three strain gauges attached to each rebar at the upper, middle, and lower parts. A total of four strain gauges were attached to the top, middle, and bottom of the concrete column. One gauge was horizontally attached at the top and bottom, and the other two were attached vertically and horizontally in the center, with four sets of such arrangement around the specimen. The specific positions of the strain gauges are shown in Figure 4.

## 3. Test Results and Analysis

### 3.1. Electrochemical Research on Steel Rebar Corrosion

The OCP is a type of mixed potential at which the anodic reaction rate equals the cathodic reaction rate, and the total net current is zero [55]. For the anodic reaction, iron is oxidized to iron ions; for the cathodic reaction, oxygen is reduced to generate hydroxide ions [56]. The OCP generally determined the corrosion state of the tested steel rebar. According to the test results, all samples had OCP values between −0.4 V and −0.7 V.

**Figure 5:**
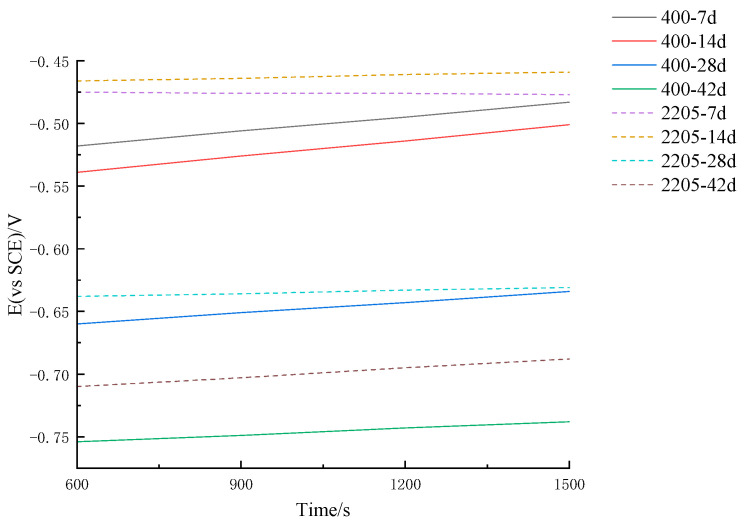
Tracking results of open circuit potentials for two kinds of steel bars at 5% Cl^−^ concentration.

Figure 5 shows the tracking results of the open-circuit potentials of ordinary steel bar HRB400 and duplex stainless steel S2205 with 5% Cl^−^ concentration in the simulated seawater environment. The same concentration had a noticeable influence on the open-circuit potentials of two different steel bars, and the open-circuit potentials of both steel bars shifted negatively.

**Figure 6:**
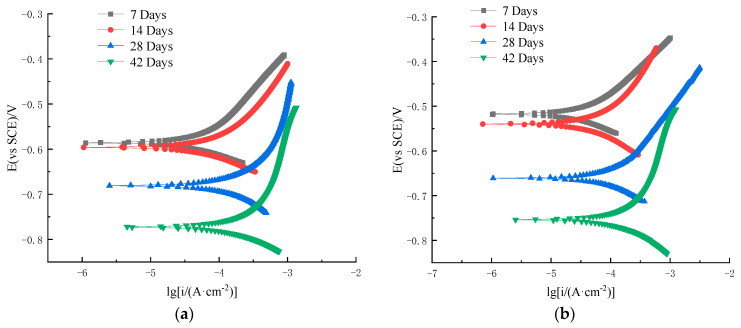
Polarization curve of HRB400 ordinary steel rebar in simulated seawater: (**a**) 3.5% Cl^−^ solution; (**b**) 5% Cl^−^ solution.

**Figure 7:**
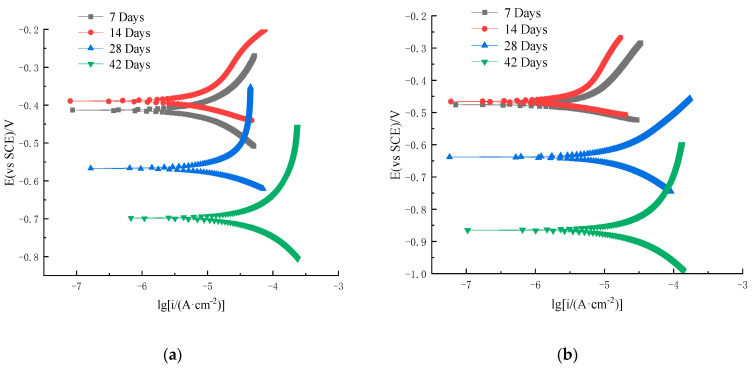
Polarization curve of S32205 steel rebar in simulated seawater: (**a**) 3.5% Cl^−^ solution; (**b**) 5% Cl^−^ solution.

Figure 6 and Figure 7 show the polarization curves of the HRB400 ordinary steel rebar and S32205 duplex stainless steel rebar in simulated seawater solutions, respectively. The polarization curves are based on the current density as the horizontal coordinate and the electrode potential as the vertical coordinate. The experimental method was designed to gradually increase the polarization of the working electrode from the self-corrosion potential at a certain potential scanning rate to a specific anodic current density value. Afterward, the working electrode is reverse-polarized at a certain scanning rate, continuously lowering its potential until reaching a self-corrosion potential, thus obtaining a curve describing the potential–current density relationship. The polarization curve can be measured by experimental methods. The analytical study of polarization curves is a basic method for explaining metal corrosion, revealing mechanisms, and exploring control pathways.

It can be seen from Figure 7a,b that the anodic curves at different chloride ions were passivated when the potential was between −0.35 V and −0.7 V and between −0.45 V and 0.9 V, respectively. These results indicate that as the concentration of chloride ions increased, the self-corrosion potential of S32205 duplex stainless steel decreased. The density of the corrosion current increased slowly, and the polarization curve tended to move to the lower right corner. This process was accompanied by a decrease in the slope of the anodic polarization curve, indicating the generation of passivation films in simulated seawater for HRB400 ordinary steel rebars and S32205 duplex stainless steel. As the activity increased, corrosive pitting increased with chloride ion concentration, and the formed passivation film was susceptible to damage by chloride ions. As a result, the corrosion rate of the steel rebar increased, and the corrosion resistance decreased. The decrease in the slope of the anodic polarization curve was gradual, indicating that the damage by chloride ions to the passivation film of the steel rebar was corrosion damage accumulation instead of instantaneous breakdown. The passivation film was punctured when the corrosion damage accumulated to a certain level.

Based on the test results of day 7 and day 14 in Figure 6 and Figure 7, the variation of the polarization curve of the S32205 duplex stainless steel rebar differed from that of the HRB400 ordinary steel rebar. At the beginning of the test, the corrosion potential slightly increased, indicating that the ability of duplex stainless steel rebar to maintain the passivation state was significantly stronger than that of ordinary steel rebar. With the erosion of chloride ions, the polarization curve of the duplex stainless steel rebar gradually shifted to the right, and the corrosion level slightly increased. Furthermore, the slope of the anodic polarization curve gradually decreased, and the reaction resistance decreased, with higher corrosion rates indicating weaker corrosion resistance. Compared to the anodic polarization curve of ordinary steel rebar, that of duplex stainless steel rebar was steeper, indicating that even if the duplex stainless steel specimens were in a more severe corrosion environment, the stability of their passivation film was better than that of carbon steel passivation film. Based on these analyses, the chloride corrosion resistance of the S32205 duplex stainless steel rebar is significantly higher than that of the HRB400 ordinary steel rebar.

Table 2 presents the fitting data of the polarization curve for the test steel rebar in simulated seawater. The corrosion current can be obtained by fitting the polarization curve. The data from simulated seawater solutions showed that the self-corrosion current increased with extended corrosion time, and the increasing trend of self-corrosion current gradually accelerated. Changes in the self-corrosion current indicated that the formed corrosion products prevented corrosion and then slowly intensified it. At the beginning of the process, the anodic slope (βa) of HRB400 ordinary steel rebar and S32205 duplex stainless steel decreased and then increased, indicating a high resistance of the anodic dissolution process. The seawater environment in concrete formed a dense passivation film on the surface of the steel rebar, hindering the occurrence of anodic reactions.

According to the S32205 duplex stainless steel data, the self-corrosion current gradually increased with the extension of corrosion time, indicating that the corrosion of the steel rebar intensified with the extension of immersion time. Compared to HRB400 ordinary steel rebar, the self-corrosion current of S32205 was very small, indicating that S32205 duplex stainless steel had good corrosion resistance in a simulated seawater environment. Due to the short reaction time, a small amount of corrosion product film formed on the surface of the sample, resulting in a small corrosion current density during the corrosion process. The corrosion product film formed in a short period was relatively loose, increasing the contact area between the medium and the substrate, thus accelerating the corrosion of the steel rebar to a certain extent. In addition, the test data showed that with the extension of corrosion time, the self-corrosion current gradually increased. The generated corrosion product film was loose and unprotective, further exacerbating corrosion, with self-corrosion currents increasing faster.

**Figure 8:**
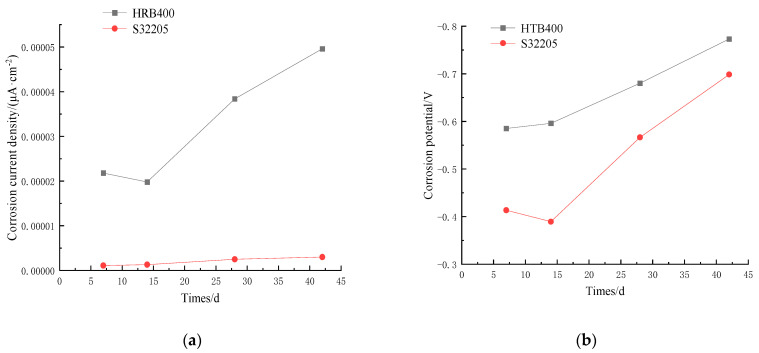
Corrosion current density and corrosion potential changes of steel rebar under 3.5% Cl^−^: (**a**) corrosion current; (**b**) corrosion potential.

**Figure 9:**
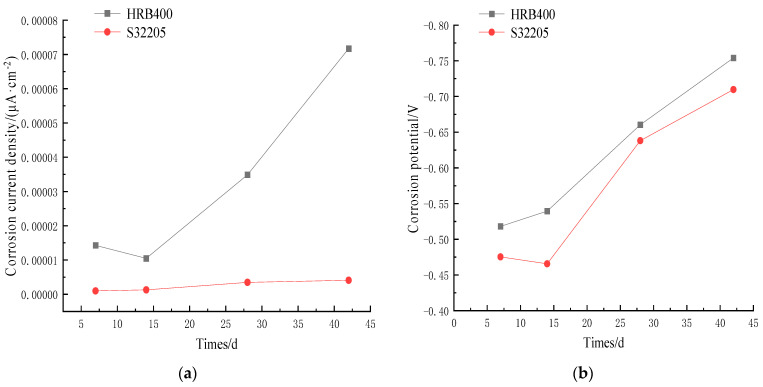
Corrosion current density and corrosion potential changes of steel rebar under 5% Cl^−^: (**a**) corrosion current; (**b**) corrosion potential.

The changes in corrosion current density (J0) and corrosion potential (E0) of HRB400 and S32205 are summarized based on Table 2 and presented in Figure 8 and Figure 9. The corrosion potential of the S32205 duplex stainless steel showed an inflection point on day 14 and began to decrease, indicating signs of corrosion. In contrast, corrosion was observed on the HRB400 steel when transferred from the water bath on day 7. As shown in Figure 8a, the self-corrosion current density of S32205 increased from 0.0000011 μA/cm^2^ to 0.0000030 μA/cm^2^ with increased corrosion time, indicating that the passivation film on the surface of the duplex stainless steel could effectively block the migration of Cl^−^ to the surface of the reinforcing steel at the initial stage of corrosion. (The corrosion current in Figure 7a ranges from 0 to 0.00001 and measures as 0.0000011).

In addition to polarization curves, EIS has also been applied to reinforced concrete to simultaneously assess the protective effect of concrete on reinforcing steel and the corrosion resistance of reinforcing steel [57,58]. The basic idea for studying electrochemical systems via EIS is as follows. An electrochemical system can be viewed as an equivalent circuit with basic components, such as resistors, capacitors, and inductors, connected in series or in parallel. The process of the equivalent circuit and the values of each component can be determined through EIS, and the electrochemical meanings of these components can be used to analyze the electrochemical system and its properties.

**Figure 10:**
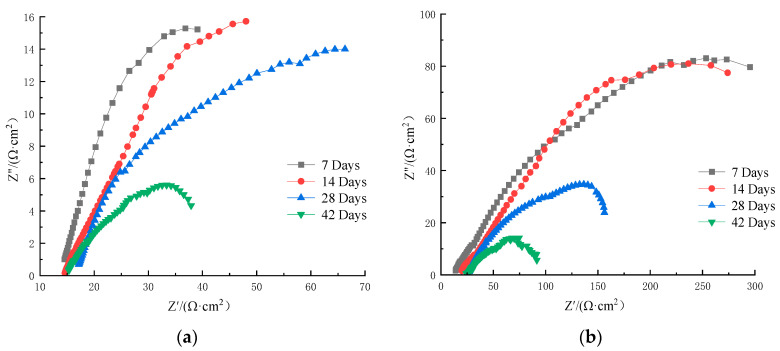
AC impedance spectrum of HRB400 rebar in simulated seawater: (**a**) 3.5% Cl^−^ solution; (**b**) 5% Cl^−^ solution.

**Figure 11:**
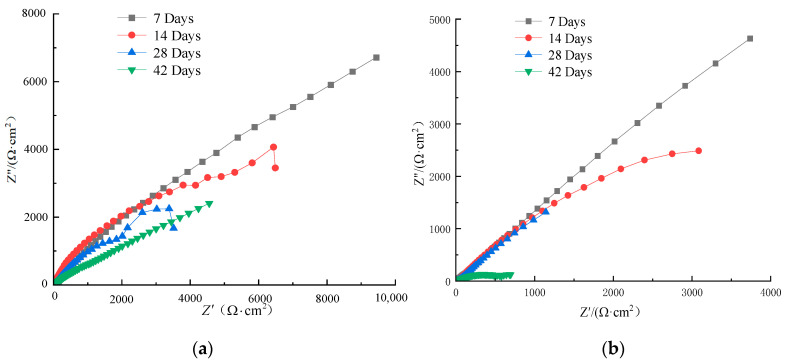
AC impedance spectrum of S32205 rebar in simulated seawater: (**a**) 3.5% Cl^−^ solution; (**b**) 5% Cl^−^ solution.

Figure 10 and Figure 11 show the AC impedance spectra of HRB400 ordinary steel rebar and S32205 duplex stainless steel rebar in simulated seawater solution, where the horizontal axis is the real part Z′ of the impedance, indicating the resistance value, while the longitudinal axis is the imaginary part Z″ of the impedance, indicating the capacitance or diffusion value.

Each point in the AC impedance spectra represents a frequency. The dense points on the left have high frequencies (hence the high-frequency region), and the dispersed points on the right have low frequencies (hence the low-frequency region). Figure 10 and Figure 11 show that the AC impedance spectra in different chloride ion solutions are composed of high and low-frequency capacitive reactance arcs, which are not semicircular. The radius of the capacitive reactance arc reflects the resistance of the passivation film and the charge transfer resistance during the electrode reaction. Generally, a greater radius of the capacitive reactance arc indicates better corrosion resistance of the metal matrix in the solution [11].

The effect of Cl^−^ on the passivation film can also be observed from the EIS of OCP. Taking the EIS of the duplex stainless steel in Figure 10 as an example, a linear curve obtained at high frequencies indicates capacitive resistance. The diameter of the capacitive reactance arc in the figure gradually decreases, indicating the formation of a passivation film on the surface of the specimen. Meanwhile, the corrosion of duplex stainless steel is aggravated, and the passivation film is damaged. Figure 11a is an equivalent circuit diagram, where the line takes a semicircular shape, indicating that the battery is a simple equivalent circuit model comprising a resistor and capacitor in parallel. In the equivalent circuit, R1 is the solution resistance, R2 represents the charge transfer resistance, and CPE is the capacitance of the double electric layer. As the Cl^−^ concentration increases, the total impedance decreases, indicating an increasing corrosion rate.

As the temperature increased, R1 decreased while CPE increased. The decrease in R2 may be attributed to the activation of the double helix surface, indicating reduced corrosion resistance of the passivation film. Meanwhile, the continuously increasing CPE indicates that the steel–concrete interface increased due to pore exposure. The prolonged immersion promotes mass transfer, which increases the concrete porosity and rebar capacitance.

As the emersion time in the simulated seawater increased, the impedance arc radius gradually decreased, indicating a decreasing polarization resistance and increasing corrosion current. As shown in Figure 10 and Figure 11, a greater numerical value of the real part of the impedance indicated a lower electrochemical reaction rate and a lower charge transfer efficiency, meaning that the corrosion resistance of S32205 duplex stainless steel was gradually enhanced. Therefore, although the corrosion resistance of S32205 duplex stainless steel was good in the early stage of corrosion, its passivation film on the surface became damaged with prolonged reaction time, decreasing its corrosion resistance. The two different reinforcing bars exhibited poor corrosion resistance in the simulated seawater solution with 5% Cl^−^. In practical applications, the corrosion resistance of S32205 duplex stainless steel in seawater is superior to that of ordinary HRB400 steel.

### 3.2. Reinforced Concrete Column Axis Compression Test

#### 3.2.1. Material Testing

Before the formal tests, tensile tests were conducted on the reinforcing bars with reference to the relevant national standards, and their elasticity modulus, yield strength, and ultimate tensile strength were measured. The mechanical properties of concrete cube test blocks were also measured, focusing on their compressive strength. See the test standards of the materials in Table 3.

In the tests, screw thread steel was used as the reinforcing bars, and HRB400 steel rebar conforming to the Code for Design of Concrete Structures (GB50010-2010) [59] was used as the rebar and stirrups. The material properties of the steel specimens were tested according to the Metallic Materials-Tensile Testing At Ambient Temperature (GB228-2002) [60] and the Metallic Materials-Test Pieces For Tensile Testing (GB6397-86) [61]. The mechanical performance indicators of the reinforcing bars are presented in Table 4.

The concrete used in the tests was cast in situ, and the same batch of C30 concrete used for the short columns was also applied to prepare concrete cubes for the compressive tests. The test methods were based on the Standard for Test Methods of Concrete Physical and Mechanical Properties (GB/T50081-2002) [62]. The concrete material test results are listed in Table 5.

#### 3.2.2. Axial Compressive Failure Analysis

**Figure 12:**
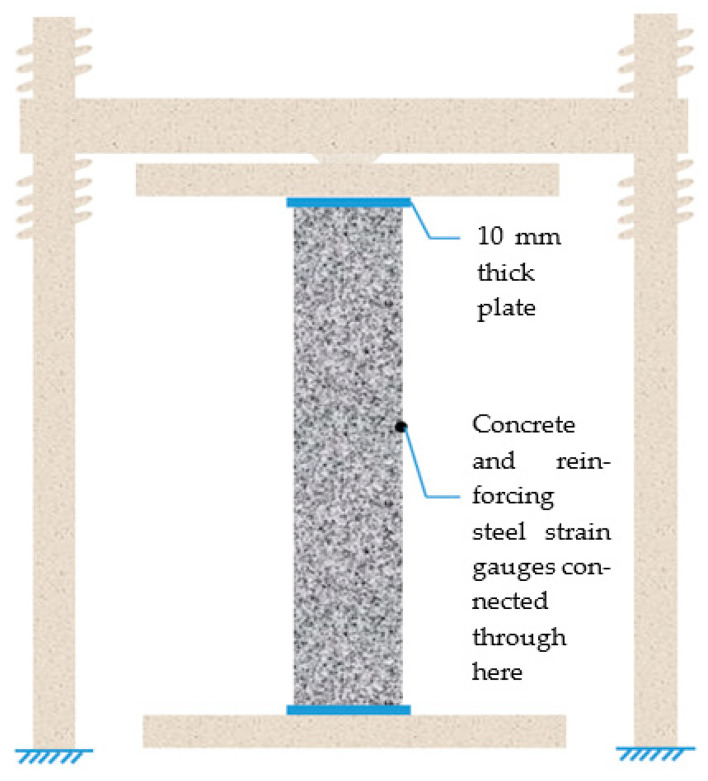
Loading device for the tests.

During the test, the specimen was placed directly on the press, and its two ends were flat-hinged. Before beginning the test, geometric alignment and physical alignment were performed. The geometric alignment was performed with the assistance of plumb lines, and the physical alignment was accomplished based on the readings of the strain gauges in the column. The specimen was preloaded before the test to check whether the equipment worked properly. Pressure-retaining loading was adopted during the formal loading, and each pressure grade was retained for 10 min. The loading pressure grade was 0.2 N_u_ (estimated ultimate load), and the loading rate was 5 kN/s. As the load reached 0.8 Nu, the loading grade was adjusted to 0.1 Nu, and the loading rate of the last loading grade was 1 kN/s. As the ultimate load was reached and retained, continuous and slow loading was adopted until specimen failure. The loading device is shown in Figure 12.

During the test, the strain data acquisition system simultaneously collected the strain readings of the longitudinal rebar, stirrups, and concrete after each load grade was stabilized. The readings were collected in a timed manner, every 0.5 s for five times, and the average of the five strain readings was recorded. The data were stored in the computer using the DH3816N Static Stress-Strain Testing and Analysis System for further analysis.

**Figure 13:**
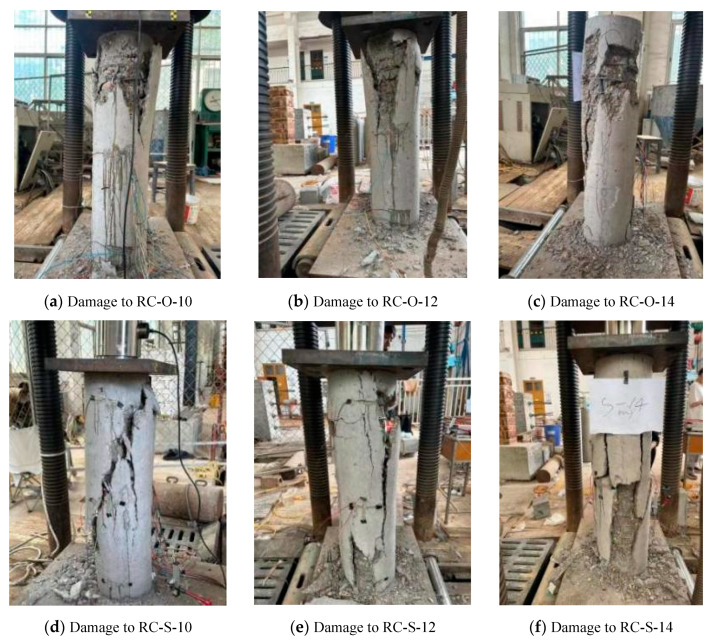
Damage to the tested column.

The compressive failure of the tested columns is shown in Figure 13. In the initial compression stage, the reinforcing bars and concrete were in the elastic working stage, where the compressive strain increased evenly. As the load reached approximately 30% of the ultimate load, fine longitudinal cracks appeared at both ends of the column along the corners. As the load increased to approximately 85% of the ultimate load, the cracks became apparent with obvious cracking sounds and began to spread and extend rapidly to the middle. When approaching the ultimate bearing capacity, the outermost concrete layer began to slowly fall off along the cracking direction as debris, with the concrete at the top and bottom of the short column damaged most severely, resulting in large debris falling off. At that time, the load decrease was significant, and the specimen lost its bearing capacity. The entire process, from cracks appearing in the concrete column to the complete failure of the specimen, was relatively short, and brittle failure characteristics were observed during the compression process. The bearing capacity test results of the reinforced concrete column are shown in Table 6.

#### 3.2.3. Comparative Analysis of Longitudinal Strain

**Figure 14:**
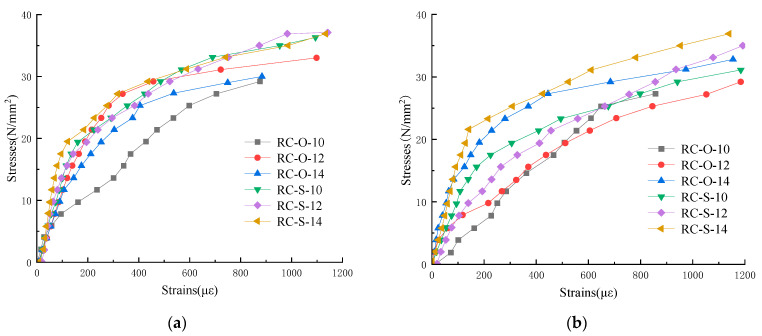
Overall stress-longitudinal strain curve: (**a**) stress-longitudinal strain diagram of concrete; (**b**) stress-longitudinal strain diagram of rebar.

The longitudinal strain in the short column axial compression test comprised the longitudinal rebar strain and longitudinal concrete strain. Figure 14 compares the stress variation curves.

It can be observed that the stress-longitudinal strain curve of each member was linearly elastic in the elastic stage. The concrete strain and longitudinal rebar strain of each member relatively coincided upon axial compression failure, with certain differences. The possible reason may be that the two ends of the specimen were not entirely flat, and the concrete and longitudinal rebar stresses were not fully synchronized during loading. The lateral expansion of the concrete during loading squeezed the longitudinal rebar, causing it to bend outward, leading to differences between the longitudinal rebar strain and longitudinal concrete strain.

#### 3.2.4. Stiffness Calculation

##### Axial Compressive Stiffness

Axial compressive stiffness refers to the ratio of deformation per unit length to stress per unit area of the structure or member under axial compression. In this paper, the stress distribution of reinforced concrete columns under axial compression was comprehensively studied based on axial compression tests, and the concept of stress was introduced to facilitate stiffness calculation. The deformation modulus of the reinforced concrete column under axis compression is expressed as follows:

(1) $$K_{2}=E\cdot A$$

where *K*_1_ represents the axial compressive stiffness of the member, *E* represents the elasticity modulus of the material, and *A* is the cross-sectional area of the member.

The test value of the short column stiffness in this study is the product of the deformation modulus test value and cross-sectional area, while the deformation modulus test value is calculated as σ/ε. Under the significant restraining effect of concrete, the longitudinal rebar strain has the least impact on the longitudinal strain of the column. Therefore, taking the longitudinal strain of the concrete as the overall strain of the reinforced concrete column is more reasonable for the test result analysis.

##### Lateral Stiffness

Lateral stiffness refers to the ratio of deformation per unit length to stress per unit area of the structure or member under horizontal pressure. To calculate the lateral stiffness, the cross-sectional shape and size of the member and the elasticity modulus of the material must be identified, which can be acquired based on the physical properties and experimental measurements of the material. The lateral stiffness equation of the member is as follows:

*K*_2_ = *E* · *I* (2)

where *K*_2_ represents the lateral stiffness of the member, *E* represents the elasticity modulus of the material, and *I* represents the cross-sectional moment of inertia. Based on this equation, the stiffness of the member under lateral stress can be calculated. A greater stiffness indicates a higher stability of the building structure and greater resistance to lateral forces.

#### 3.2.5. Stiffness Calculation

##### Axial Compressive Stiffness Theoretical and Test Value Comparison

When calculating the axial compressive stiffness test value of the reinforced concrete short column, the 18 short columns in the tests were taken as objects, and the calculations were based on Equation (1). The comparison between the theoretical and test values for the axial compressive stiffness is presented in Table 7.

In this study, the longitudinal strain of concrete was considered as its actual strain. The reinforced concrete axial compressive stiffness test results show that the test values of the two sets of short columns were greater than the theoretical calculation results. The average error was 0.47% in the RC-O group and −4.1% in the RC-S group. The axial compressive stiffness of the RC-S group was greater than that of the RC-O group.

##### Lateral Stiffness Theoretical and Test Value Comparison

Based on the lateral stiffness theory, the 18 short columns in the tests were taken as objects, and the calculations were conducted with the theoretical Equation (2). The comparison between the theoretical and test values for lateral stiffness is presented in Table 8.

The comparison between the theoretical calculation and test results suggests that the lateral stiffness derived from the physical measurements of the cross-sectional diameter of the short column and the tested elasticity modulus of the concrete prism was significantly greater than the test results. The short-column lateral stiffness test values suggest that the test results were greater than the theoretical calculation results. The average error was 0.83% in the RC-O group and −3.5% in the RC-S group. The lateral stiffness of the RC-S group is greater than that of the RC-O group.

## 4. Conclusions

Through experimental research, the corrosion of two reinforcing bars under different erosion conditions was explored, and the factors affecting the stiffness of short columns fabricated using the two steels as longitudinal rebars were investigated. The conclusions are as follows:

(1) The polarization curves of HRB400 ordinary steel and S32205 duplex stainless steel tended to move toward the lower right corner, accompanied by decreases in the slope of the anodic polarization curves. This was caused by the increasingly concentrated chloride ions around the reinforcing steel damaging the passivation film of the reinforcing steel to a certain extent. The decline in the slope of the anodic polarization curve was gradual, indicating that the damage to the passivation film of reinforcing steel by the chloride ions was not instantaneous but a process of accumulation. The corrosion rate of the S32205 duplex stainless steel in seawater is approximately 1/15 that of regular HRB400 steel.
(2) As observed on the polarization curve on days 7 and 14, the polarization curve of S32205 duplex stainless steel rebar had different variation trends than HRB400 ordinary steel rebar. The slight increase in the corrosion potential at the initial stage of the experiment indicated a significantly greater ability of duplex stainless steel rebar to maintain a passive state than regular steel rebar. A comparison between the polarization curves of the two different reinforcing bars suggested that the duplex stainless steel rebar exhibits a larger slope and a steeper anodic polarization curve. Therefore, the passivation film of duplex stainless steel rebars is more stable than that of common carbon steel rebars, even in more harsh corrosion environments.
(3) The AC impedance spectrum showed that the impedance arc radius gradually decreased with increased corrosion time, indicating a decreasing polarization resistance and increasing corrosion current. A greater numerical value of the real part of the impedance indicated a lower electrochemical reaction rate and charge transfer efficiency. Therefore, the corrosion resistance of the S32205 duplex stainless steel in seawater is superior to that of ordinary HRB400 steel.
(4) Two groups of reinforced concrete short columns with different longitudinal rebars were designed and tested, and the comparative analysis of test and theoretical calculation results showed that the axial compressive stiffness and lateral stiffness test results were greater than the theoretical calculations. Specifically, the average error of axial compressive stiffness was 0.47% in the RC-O group and −4.1% in the RC-S group. The stiffness test values for the 12 mm longitudinal rebar short columns were optimal. The average error of lateral stiffness was -0.83% in the RC-O group and −3.5% in the RC-S group. In general, the stiffness of the RC-S group is greater than that of the RC-O group.

## Figures and Tables

**Table 1:** Chemical composition of test steel (mass fraction %).

Element	C	Si	Mn	P	S	Ceq	Ni	Cr	Mo	N
S32205	0.019	0.56	1.07	0.018	0.018	—	5.59	22.36	3.16	0.14
HRB400	0.25	0.45	1.4	0.28	0.17	0.49	—	—	—	—

**Table 2:** Polarization curve fitting data for HRB400 and S32205 in simulated seawater solutions.

Specimen	Simulation Solution	t/d	βa/(mV·dec^−1^)	βc/(mV·dec^−1^)	J0/(μA·cm^−2^)	E0/V
HRB400	3.5% Cl^−^	7	34.84	−30.33	0.0000218	−0.5852
		14	34.16	−31.17	0.0000198	−0.5959
		28	32.46	−33.42	0.0000384	−0.6802
		42	37.31	−29.76	0.0000496	−0.7731
	5% Cl^−^	7	34.69	−30.27	0.0000143	−0.5181
		14	33.19	−32.54	0.0000105	−0.5394
		28	35.88	−33.67	0.0000349	−0.6604
		42	35.87	−30.89	0.0000717	−0.7540
S32205	3.5% Cl^−^	7	35.22	−31.96	0.0000013	−0.4135
		14	37.90	−28.45	0.0000011	−0.3894
		28	36.93	−29.91	0.0000025	−0.5666
		42	34.93	−31.95	0.0000030	−0.6985
	5% Cl^−^	7	36.94	−28.65	0.0000013	−0.4754
		14	39.31	−27.10	0.0000010	−0.4657
		28	34.12	−32.50	0.0000035	−0.6381
		42	33.53	−32.69	0.0000041	−0.7098

**Table 3:** Test standards of materials.

Material Standard Value (N/mm^2^)		
HRB400 steel bar	yield strength	400
	ultimate strength	540
	modulus of elasticity	2.0 × 10^5^
Concrete test block	axial compressive strength	2.01
	axial compressive strength	20.1
	modulus of elasticity	3.0 × 10^4^

**Table 4:** Mechanical performance indicators of reinforcing bars.

Material Type		Rebar Diameter (mm)	Yield Strength (N/mm^2^)	Ultimate Tensile Strength (N/mm^2^)
HRB400	longitudinal rebar	10	339.26	399.13
		12	359.24	438.94
		14	395.18	465.58
	stirrup	6	331.41	424.00
S32205	longitudinal rebar	10	609.10	716.59
		12	683.08	803.60
		14	563.90	583.97
	stirrup	6	554.35	650.42

**Table 5:** Concrete material properties test results.

Concrete Type	Specimen Size (mm)	Ultimate Load (kN)	Compressive Strength (N/mm^2^)	Elasticity Modulus (N/mm^2^)
C30	150 × 150 × 150	666.48	29.71	-
	150 × 150 × 300	690.82	30.70	3.07 × 10^4^

**Table 6:** Comparative analysis of the theoretical and test values of the ultimate bearing capacity of reinforced concrete columns.

Code	Theoretical Mean /kN	Test Mean /kN	Test Value/Theoretical Value
RC-O-10	1349.81	1430.80	1.06
RC-S-10	1635.64	1870.67	1.14
RC-O-12	1450.62	1646.27	1.13
RC-S-12	1822.41	1996.30	1.09
RC-O-14	1569.49	1730.13	1.10
RC-S-14	1817.07	2213.97	1.21

**Table 7:** Axial compressive stiffness comparison.

Column Code	Theoretical Value (N)	Test Value (N)	Difference Percentage (%)
RC-O-10	1.54 × 10^9^	1.51 × 10^9^	2.0
RC-S-10		1.59 × 10^9^	−3.1
RC-O-12		1.57 × 10^9^	−1.9
RC-S-12		1.63 × 10^9^	−5.5
RC-O-14		1.52 × 10^9^	1.3
RC-S-14		1.60 × 10^9^	−3.7

**Table 8:** Lateral stiffness comparison.

Column Code	Theoretical Value (N·mm^2^)	Test Value (N·mm^2^)	Difference Percentage (%)
RC-O-10	6.32 × 10^12^	6.16 × 10^12^	2.6
RC-S-10		6.51 × 10^12^	−2.9
RC-O-12		6.42 × 10^12^	−1.5
RC-S-12		6.65 × 10^12^	−5.0
RC-O-14		6.23 × 10^12^	1.4
RC-S-14		6.55 × 10^12^	−2.6

## Data Availability

All data used to support the study are included within the article.
